# Utilisation of HIV pre-exposure prophylaxis and preferences of alternative long-acting modalities among men who have sex with men in Hong kong: a cross-sectional study

**DOI:** 10.1186/s12889-025-26025-5

**Published:** 2025-12-26

**Authors:** Tsz Ho Kwan, Denise Pui Chung Chan, Shui Shan Lee

**Affiliations:** 1 Jockey Club School of Public Health and Primary Care, The Chinese University of Hong Kong, Shatin, Hong Kong; 2 S.H. Ho Research Centre for Infectious Diseases, The Chinese University of Hong Kong, Shatin, Hong Kong; 3 Stanley Ho Centre for Emerging Infectious Diseases, The Chinese University of Hong Kong, Shatin, Hong Kong

**Keywords:** Long-acting PrEP, Men who have sex with men, Injectable, Pre-exposure prophylaxis

## Abstract

**Background:**

Pre-exposure prophylaxis (PrEP) has changed the paradigm of HIV prevention. A majority of men who have sex with men (MSM) were using oral tablets; meanwhile alternative long-acting modalities have been and are being developed. We aimed to assess the prevalence of PrEP use and the preferences of potential long-acting PrEP in the MSM community in Hong Kong, an Asian Pacific city where free or subsidised PrEP is not available.

**Methods:**

A cross-sectional study was conducted using an online self-administered questionnaire. Eligible participants were adult MSM without HIV infection who normally resided in Hong Kong. Historical and current PrEP use, and preferences of using long-acting injectable for PrEP were tested against sociodemographic and sexual behavioural factors using chi-squared test and multivariable logistic regression model.

**Results:**

Of the 437 responses collected, 17% had ever used PrEP and 16% were currently using PrEP. Current PrEP users were more likely to have a higher income level (adjusted odds ratio [aOR] 2.11, *p* = 0.04), have engaged in chemsex (aOR 4.02, *p* < 0.01), have a non-regular male sex partner (aOR 2.66, *p* = 0.02), have a male sex partner living with HIV (aOR 3.60, *p* < 0.01), and be inclined to have condomless sex in the future (aOR 6.86, *p* < 0.01). Among ever users, MSM who discontinued PrEP had a lower sexual risk than current users, such as recent history of chemsex (OR 0.22, *p* < 0.01) and inclination to have condomless sex (OR 0.15, *p* < 0.01). Long-acting injectables were accepted by 27% MSM, who were more likely to be current PrEP users (odds ratio [OR] 2.35, *p* < 0.01), concerned about efficacy (OR 1.66, *p* = 0.03), and expecting to use PrEP for more than 2 years in the future (OR 9.69, *p* < 0.01).

**Conclusion:**

The higher prevalence of PrEP among MSM indicated an increased awareness over the past decade. However, its promotion could have been hindered by the absence of a publicly funded or subsidised programme. While PrEP users had a higher sexual risk, the utilisation pattern would be affected by the dynamicity of sexual activities. Long-acting injectables had a moderate acceptance rate. Current PrEP users and those anticipated long-term users could be prioritised for its promotion.

**Supplementary Information:**

The online version contains supplementary material available at 10.1186/s12889-025-26025-5.

## Introduction

HIV pre-exposure prophylaxis (PrEP) is a key element in the HIV prevention toolbox. Daily oral fixed-dose combination tablets demonstrated a significant efficacy in lowering the risk of HIV acquisition in various populations, including men who have sex with men (MSM) [1], a population disproportionately affected by the HIV epidemic [2]. An alternative dosage option, on-demand PrEP, allows MSM to take the tablets orally before and after an episode of sexual activity with a comparable efficacy to better suit their sexual activity pattern [3]. A previous study also showed that a high level of prevention-effective adherence could be achieved by either regimen option [4, 5]. The next generation oral tablet has been shown to be non-inferior with less effect on the kidneys and bones [6]. While the overall coverage of PrEP has gradually increased, the proportion of PrEP-using MSM varies geographically [7].

Currently, other advancements in PrEP are emerging, which included a bimonthly [8] and biannual (ClinicalTrials.gov ID: NCT04925752 and the upcoming PURPOSE 5 trial) long-acting injectable, which expands the options from oral drugs to injections [8]. A recent study showed that the bimonthly injectable was superior to daily oral tablets [9]. It could also be a useful option for those who face difficulties in following the dosage schedule [10], or need to hide the tablets away for avoiding stigmas and protecting privacy [11]. However, not all MSM are willing to receive regular injections. Major barriers could include fear of needles [12], and inconvenience due to the more frequent follow-up visits [13]. In addition, there are some other modalities for PrEP delivery in the research and development pipeline [14], including vaginal rings, implants, and rectal gel, all aiming to provide more options that could address individual preferences and needs.

In many places including Hong Kong, where PrEP is not publicly available, the source of PrEP relies heavily on overseas clinics and services [15]. This may result in structural barriers to the access of PrEP in the MSM community, especially for those who have a lower income level. Similar to other places, there are also stigmas and labelling issues against PrEP [16]. The landscape of PrEP use in the community against this complicated background is understudied. This study aims to determine the current PrEP use pattern in the Hong Kong MSM community, and to assess their preferences of different modalities of PrEP that may become available in the future.

## Methods

A cross-sectional study was conducted between 1 Dec 2022 and 31 May 2023. Eligible participants were assigned male at birth, had ever had sex with a male, aged 18 years or above, were normally residing in Hong Kong, and self-reported not HIV-positive. A sample size of 385 was set to determine the prevalence of PrEP uptake at a 95% confidence level and 5% margin of error with an expected prevalence of 50% to yield a maximum sample size. Promotional materials were posted in online platforms frequently used by the MSM community. Voluntary participants were invited to complete an online, open, self-administered questionnaire after giving online informed consent. Upon completion, they would be given HK$50 (HK$7.8 ≈ US$1) cash voucher for compensating their time. Given the multi-part nature of the survey, adaptive questionnaire was used to reduce the number and complexity of the questions by skipping unnecessary questions based on previous answers. As participants could go back to the previous page, no review step was designed towards the end of the survey. Duplicate entries were defined as entries with the same IP address within 24 h. Only the first entry was retained in the analysis. No cookies or log file analysis were used to identify multiple entries. Completeness check was in place before the participant could proceed to the next page or submit.

The self-developed questionnaire consisted of five parts: (a) sociodemographics, (b) sexual behaviour history, (c) perceived HIV/sexually transmitted infection (STI) risks and STI history, (d) PrEP awareness and use, and (e) perceptions about different PrEP modalities (Additional file 1). Sociodemographic factors enquired included age, ethnicity, education level, income level, and self-perceived body image types [17]. Recent (past six months) sex seeking and sexual practices, namely sexual role, condom use, types of sex partners, chemsex, and group sex, were enquired. Perceived HIV and STI risks were separately measured using a four-level Likert scale: no risk, low risk, medium risk, and high risk. Respondents were also asked about the perceived HIV status of their partners, their inclination to have condomless sex in the future six months, and history of STI diagnosis in the past six months. Regarding PrEP, they were asked about their awareness and if they had ever used or were currently using PrEP. Preferred regimen of oral PrEP was enquired, along with the reasons for choosing the regimen. Acceptable price and potential duration for PrEP use were enquired. Preferences of long-acting modalities in the development pipeline other than oral tablets, including injectable drugs, vaccines, implants, and rectal gels, were asked. The preferred schedule of injectable was also enquired.

Factors associated with current PrEP use were tested against sociodemographic, sexual behavioural factors, and HIV/STI risk perception [17]. Preferred alternative long-acting PrEP modalities were dichotomised before statistical analysis. Binary dependent outcome variables were tested against categorical and continuous factors using chi-squared and Mann-Whitney U test respectively. Variables with a p value < 0.05 in bivariate analysis were inputted to a binary logistic regression model for adjusting potential confounders. Associations between consideration factors of choosing a PrEP service provider and different potential types of such providers were similarly tested. This study was approved by The Chinese University of Hong Kong Survey and Behavioural Research Ethics Committee (Ref. No. SBRE-21-0470). The study complied with the Declaration of Helsinki. The reporting of the results followed the CHERRIES checklist [18].

## Results

### Characteristics of participating MSM

Of 760 who clicked through the consent form, 596 completed the eligibility screening and were eligible, and 458 completed the survey. After removing 21 duplicate entries, the sample size for analysis was 437. The median age of the participating MSM was 31 year (interquartile range 27–35 years) (Table 1). The majority (95%) was locally residing ethnic Chinese, and had attained university education level or above (71%). About one-third had a monthly income of over HK$30,000 (≈ US$3837). In the past six months, about 70% had sought new male sex partners; a smaller proportion engaged in chemsex (14%) and group sex (26%). Most (90%) participants were aware of PrEP. Of 96 (22%) MSM who had ever used PrEP, 71% were still using it. While 82% MSM had had anal intercourse in the past six months, only 33% had always used a condom. Meanwhile, half (50%) of the MSM were inclined to have condomless anal intercourse in the future six months. About 13% perceived that they had a male sex partner living with HIV; some 14% and 11% self-perceived to have no risk of HIV and STI, respectively.

**Table 1:** Characteristics of the study sample (*N* = 437)

	*n* (%)
**Sociodemographic factors**	
Median age, years (IQR)	31 (27–35)
Locally residing ethnic Chinese	417 (95%)
University education level or above	312 (71%)
Monthly income > HK$30,000	145 (33%)
**Body image type**	
Muscular	42 (10%)
Sporty	68 (16%)
Bear	34 (8%)
Meaty	141 (32%)
**Sexual behaviour profile in the past six months**	
Sought male sex partners	305 (70%)
Engaged in anal intercourse	357 (82%)
Always used a condom when having anal intercourse (*N* = 357)	118 (33%)
Engaged in chemsex	60 (14%)
Had group sex	113 (26%)
Diagnosed with an STI	26 (6%)
Had a regular male sex partner	170 (39%)
Had a non-regular male sex partner	243 (56%)
Had a romantic male sex partner	179 (41%)
**PrEP**	
Ever heard of PrEP	393 (90%)
Ever used PrEP	96 (22%)
Currently using PrEP	68 (16%)
**HIV/STI risk perception**	
Inclined to have condomless anal intercourse in the future six months	218 (50%)
Perceived to have a sex partner living with HIV	58 (13%)
Perceived to have no HIV risk	59 (14%)
Perceived to have no STI risk	47 (11%)

### Factors associated with current PrEP use

Current PrEP users, compared with MSM not currently using PrEP, were older (*p* < 0.01), had a higher monthly income (*p* < 0.01), were more likely to have attained university level education or above (*p* = 0.01), and were self-identified to be muscular (*p* = 0.01) or sporty (*p* = 0.02) type (Table 2). In term of sexual behaviours in the past six months, current PrEP users had higher odds of seeking new male sex partners (odds ratio [OR] 3.28, 95% confidence interval [CI] 1.57–6.83, *p* < 0.01), having condomless anal intercourse (CLAI) (OR 9.35, IQR 4.17–20.99, *p* < 0.01), engaging in chemsex (OR 8.11, 95% CI 4.43–14.85, *p* < 0.01) and group sex (OR 3.96, 95% CI 2.31–6.77, *p* < 0.01), having a regular (OR 3.04, 95% CI 1.78–5.19, *p* < 0.01) or non-regular (OR 3.67, 95% CI 1.97–6.84, *p* < 0.01) male sex partner, and inclining to have CLAI in the future six months (OR 1.02, 95% CI 4.66–21.55, *p* < 0.01). They were also more likely to believe to have a male sex partner living with HIV (*p* < 0.01), and have a higher perceived risk of HIV (*p* = 0.02) and STI (*p* = 0.01). Variables included in the final multiple logistic regression model were monthly income (adjusted OR [aOR] 2.11, 95% CI 1.02–4.36), chemsex engagement (aOR 4.02, 95% CI 1.73–9.36), having a non-regular male sex partner (aOR 2.66, 95% CI 1.17–6.04), inclination to have CLAI (aOR 6.86, 95% CI 2.56–18.38), and having an HIV-positive male sex partner (aOR 3.60, 95% CI 1.50–8.65).

**Table 2:** Comparison between current PrEP users and non-users (*N* = 437)

	Currently using PrEP (*n* = 68)	Not currently using PrEP (*n* = 369)	OR (95% CI)	*P* value	Adjusted OR (95% CI)	*P* value
Median age, years (IQR)	34 (29–40)	31 (27–34)	N/A	< 0.01	1.02 (0.98–1.07)	0.32
Monthly income over HK$30,000	37 (54%)	108 (29%)	2.88 (1.70–4.89)	< 0.01	2.11 (1.02–4.36)	0.04
Attained university level education or above	58 (85%)	254 (69%)	2.63 (1.30–5.32)	0.01	2.51 (0.96–6.59)	0.06
Body image type: muscular	12 (18%)	30 (8%)	2.42 (1.17–5.01)	0.01	2.03 (0.70–5.87)	0.19
Body image type: sporty	17 (25%)	51 (14%)	2.08 (1.11–3.88)	0.02	1.57 (0.66–3.71)	0.31
Body image type: bear	6 (9%)	28 (8%)	1.18 (0.47–2.96)	0.73	-	-
Body image type: meaty	19 (28%)	122 (33%)	0.79 (0.44–1.39)	0.41	-	-
Had CLAI in the past 6 months	61 (90%)	178 (48%)	9.35 (4.17–20.99)	< 0.01	1.72 (0.61–4.84)	0.30
Engaged in chemsex in the past 6 months	29 (43%)	31 (8%)	8.11 (4.43–14.85)	< 0.01	4.02 (1.73–9.36)	< 0.01
Engaged in group sex in the past 6 months	35 (51%)	78 (21%)	3.96 (2.31–6.77)	< 0.01	1.07 (0.49–2.36)	0.87
Sought male sex partners in the past 6 months	59 (87%)	246 (67%)	3.28 (1.57–6.83)	< 0.01	1.10 (0.39–3.10)	0.86
Had a regular male sex partner in the past 6 months	42 (62%)	128 (35%)	3.04 (1.78–5.19)	< 0.01	1.42 (0.68–2.97)	0.35
Had a non-regular male sex partner in the past 6 months	54 (79%)	189 (51%)	3.67 (1.97–6.84)	< 0.01	2.66 (1.17–6.04)	0.02
Had a romantic male partner in the past 6 months	27 (40%)	152 (41%)	0.94 (0.55–1.59)	0.82	-	-
Diagnosed with an STI in the past 6 months	12 (18%)	14 (4%)	5.40 (2.16–13.32)	< 0.01 *	2.88 (0.98–8.43)	0.05
Perceived no HIV risk	3 (4%)	56 (15%)	0.26 (0.08–0.85)	0.02	0.57 (0.09–3.47)	0.54
Perceived no STI risk	1 (1%)	46 (12%)	0.10 (0.01–0.77)	0.01	0.49 (0.05–5.11)	0.55
Inclined to have CLAI in the coming 6 months	60 (88%)	158 (43%)	10.02 (4.66–21.55)	< 0.01	6.86 (2.56–18.38)	< 0.01
Perceived to have a male sex partner living with HIV	24 (35%)	34 (9%)	5.37 (2.92–9.89)	< 0.01	3.60 (1.50–8.65)	< 0.01

* By Fisher’s exact test

Among MSM who had ever used PrEP, compared with current PrEP users, those who were no longer using PrEP (*n* = 28) were less likely to have an HIV-positive partner (OR 0.07, 95% CI 0.01–0.53, *p* < 0.01), or a regular male sex partner (OR 0.34, 95% CI 0.14–0.86, *p* = 0.02). In the past six months they were also less likely to engage in chemsex (OR 0.22, 95% CI 0.07–0.72, *p* < 0.01), and condomless sex (OR 0.15, 95% CI 0.05–0.45, *p* < 0.01); and they were less likely to be inclined to have condomless sex in the coming six months (OR 0.12, 95% CI 0.04–0.33, *p* < 0.01).

### Perceptions about long-acting injectables

Among the four alternative modalities of PrEP in the pipeline, vaccine was the most accepted (*n* = 247, 57%), followed by injectable drugs (*n* = 120, 27%). Implants (*n* = 70, 16%) and rectal gel (*n* = 67, 15%) had a low acceptance rate. Some 80 (18%) chose injectable drugs as the most preferred modality over alternative long-acting options and non-use. Current PrEP users were more likely to prefer injectable drugs (OR 4.43, 95% CI 2.59–7.59, *p* < 0.01) (Table 3). Those who preferred injectables were more likely to have been diagnosed with an STI (OR 3.63, 95% CI 1.60–8.25, *p* < 0.01), have a regular male sex partner (OR 1.75, 95% CI 1.07–2.85, *p* = 0.02), have engaged in group sex (OR 2.60, 95% CI 1.56–4.33, *p* < 0.01), and have had condomless anal intercourse (OR 1.69, 95% CI 1.02–2.80, *p* = 0.04) in the past six months. They were also more likely to perceive to have a male sex partner living with HIV (OR 2.30, 95% CI 1.24–4.27, *p* < 0.001) and some risk of HIV acquisition (OR 2.67, 95% CI 1.03–6.92, *p* = 0.04). After adjusting for confounders, significant factors in the multiple regression model for long-acting injectable preference included engagement in group sex (aOR 1.05, 95% CI 1.86–3.31) and STI diagnosis (aOR 1.01, 95% CI 2.49–6.16).

**Table 3:** Associating factors of preferences for long-acting injectable PrEP (*N* = 437)

	Preferred injectables (*n* = 80)	Did not prefer injectables (*n* = 357)	OR (95% CI)	*P* value	Adjusted OR (95% CI)	*P* value
Median age, years (IQR)	33 (28–36)	31 (27–35)	N/A	0.05	-	-
Monthly income over HK$30,000	33 (41%)	112 (31%)	1.54 (0.93–2.53)	0.09	-	-
Attained university level education or above	61 (76%)	251 (70%)	1.36 (0.77–2.38)	0.29	-	-
Body image type: muscular	7 (9%)	35 (10%)	0.88 (0.38–2.06)	0.77	-	-
Body image type: sporty	18 (23%)	50 (14%)	1.78 (0.97–3.26)	0.06	-	-
Body image type: bear	7 (9%)	27 (8%)	1.17 (0.49–2.79)	0.72	-	-
Body image type: meaty	29 (36%)	112 (31%)	1.24 (0.75–2.07)	0.40	-	-
Had CLAI in the past 6 months	52 (65%)	187 (52%)	1.69 (1.02–2.8)	0.04	0.42 (0.85–1.74)	0.66
Engaged in chemsex in the past 6 months	20 (25%)	40 (11%)	2.64 (1.44–4.83)	< 0.01	0.63 (1.31–2.72)	0.47
Engaged in group sex in the past 6 months	34 (43%)	79 (22%)	2.60 (1.56–4.33)	< 0.01	1.05 (1.86–3.31)	0.03
Sought male sex partners in the past 6 months	62 (78%)	243 (68%)	1.62 (0.91–2.86)	0.10	-	-
Had a regular male sex partner in the past 6 months	40 (50%)	130 (36%)	1.75 (1.07–2.85)	0.02	0.70 (1.20–2.06)	0.51
Had a non-regular male sex partner in the past 6 months	49 (61%)	194 (54%)	1.33 (0.81–2.18)	0.26	-	-
Had a romantic male partner in the past 6 months	33 (41%)	146 (41%)	1.01 (0.62–1.66)	0.95	-	-
Diagnosed with an STI in the past 6 months	11 (14%)	15 (4%)	3.63 (1.63–8.25)	< 0.01	1.01 (2.49–6.16)	0.047
Perceived no HIV risk	5 (6%)	54 (15%)	0.37 (0.14–0.97)	0.04	0.17 (0.45–1.20)	0.011
Perceived no STI risk	4 (5%)	43 (12%)	0.38 (0.13–1.1)	0.07	-	-
Inclined to have CLAI in the coming 6 months	49 (61%)	169 (47%)	1.76 (1.07–2.89)	0.02	0.74 (1.45–2.87)	0.28
Perceived to have a male sex partner living with HIV	18 (23%)	40 (11%)	2.30 (1.24–4.27)	0.01	0.88 (1.76–3.52)	0.11
Current PrEP user	21 (26%)	47 (13%)	2.35 (1.31–4.21)	< 0.01	0.54 (1.10–2.25)	0.80

**Fig. 1:**
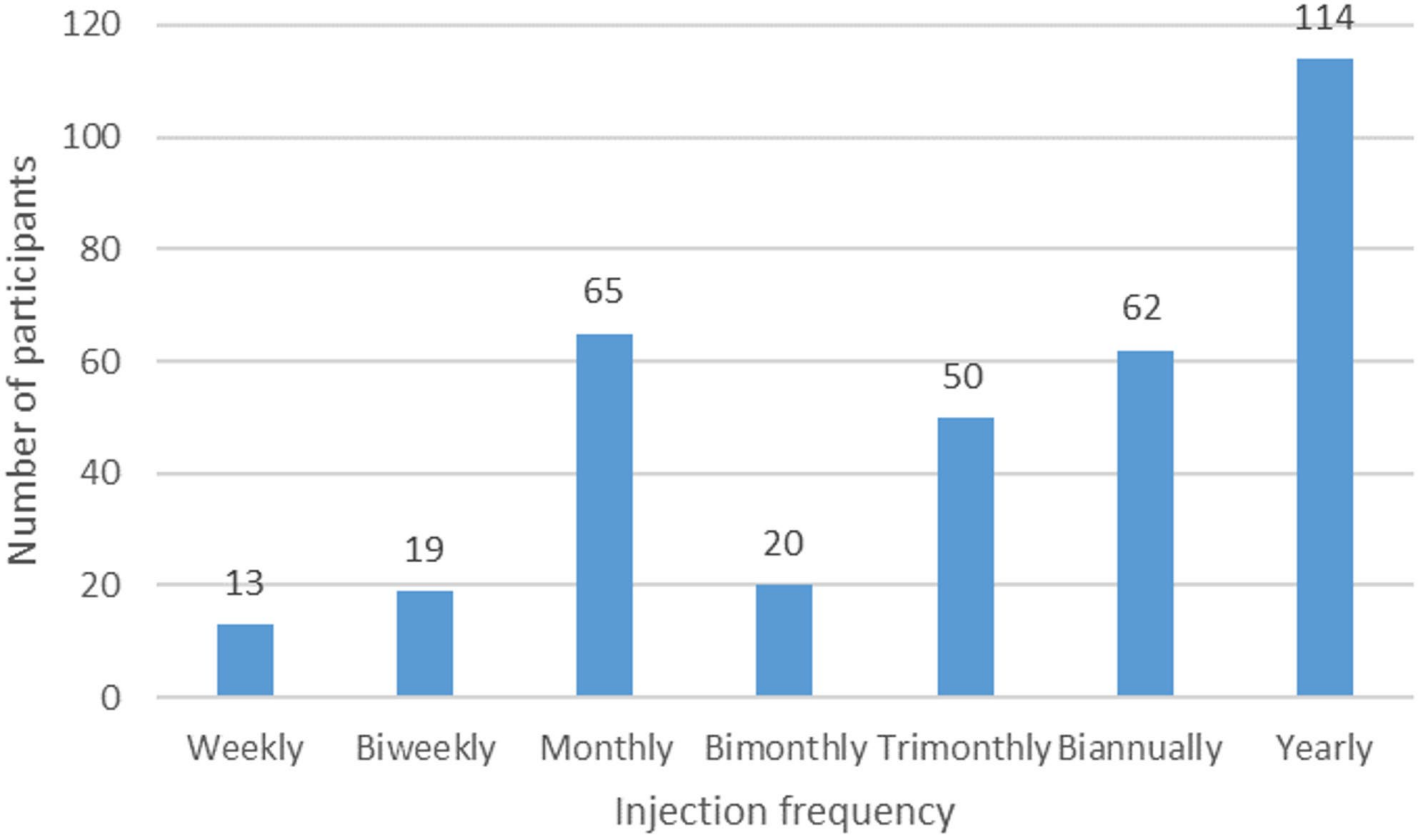
Most favoured injection frequency chosen by MSM (*N* = 343)

Some 62% considered efficacy important when choosing a PrEP option. They were more likely to accept long-acting injectables for HIV PrEP (OR 1.66, 95% CI 1.06–2.61, *p* = 0.03). Concerns about potential adverse events (71%), dosing schedule (25%), price (83%), service provider (27%), location (25%), operating hours (21%), privacy (35%), and labelling (28%) were not associated with the injectable PrEP acceptance. Those accepting long-acting injectables were more likely to be expecting to use PrEP for a longer duration of over 2 years (OR 9.69, 95% CI 5.47–17.16, *p* < 0.01). As regards the preferred schedule of the injection, a bimodal distribution was observed (Fig. 1). Of the 343 participants who would consider receiving an injection regularly for HIV prevention, among the options available, yearly was the most favoured frequency (33%), followed by monthly (19%), biannually (18%), and trimonthly (15%). The other options, including weekly, biweekly, and bimonthly, were only accepted by 4–6% of the participants.

### Association between service providers and preferences of injectables

Participants who accepted injectables for PrEP were more likely to accept receiving PrEP in sexual health clinics (OR 2.02, 95% CI 1.31–3.12, *p* < 0.01), public clinics (OR 2.67, 95% CI 1.70–4.19, *p* < 0.01), public HIV services (OR 1.94, 95% CI 1.24–3.03, *p* < 0.01), and public hospital (OR 1.69, 95% CI 1.03–2.79, *p* = 0.04). Table 4 shows the correlation between different types of service providers and their consideration factors when choosing a PrEP option. Type of service provider as a consideration factor was associated with all types of providers. Participants who preferred receiving PrEP in the private sector were concerned about all factors when choosing a PrEP option. Participants who were concerned about privacy and labelling were more likely to prefer public and private hospitals.

**Table 4:** Association between consideration factors and the type of potential PrEP service provider (*N* = 437)

	Sexual health clinics^	Public clinics^	Public hospitals^	Public HIV services^	Private clinics or hospitals	Community-based organisations
Price		2.14 (1.11–4.14)		1.88 (1.01–3.50)	2.21 (1.09–4.49)	2.31 (1.38–3.85)
Provider	1.68 (1.09–2.58)	2.11 (1.34–3.33)	2.25 (1.38–3.69)	2.01 (1.28–3.15)	1.78 (1.11–2.87)	1.70 (1.03–2.80)
Location		1.75 (1.10–2.79)	1.99 (1.20–3.28)		1.81 (1.12–2.93)	2.49 (1.43–4.33)
Operation time		2.33 (1.43–3.80)	2.16 (1.28–3.66)		2.18 (1.32–3.62)	2.55 (1.38–4.71)
Privacy			1.64 (1.02–2.64)		2.17 (1.38–3.40)	
Labelling issue			1.67 (1.02–2.74)		2.14 (1.34–3.42)	

^ Significantly associated with the acceptance of long-acting injectable PrEP

# Only significant associations (odds ratio and the respective 95% confidence interval) assessed using chi-squared test between consideration factors and the type of service providers were presented

## Discussions

Our results showed that the current prevalence of PrEP use in Hong Kong was low at 16%, though higher than the previously reported rate [15]. MSM at a higher risk of HIV acquisition were more likely to be using PrEP, and they were more likely to accept using long-acting injectable PrEP should it become available. Current PrEP use was associated with socioeconomic factors including age, income level, and education level. In Hong Kong, as PrEP is not publicly available, MSM primarily relied on private services locally or overseas which incurred some costs [15]. This created a structural barrier for those who have a lower income level to access PrEP equitably. The prevalence of PrEP use can unlikely become much higher without a subsidised programme in place locally. A previous study showed that body image type was a key attribute in sexual mixing in the MSM community [19]. MSM of certain body image types were shown to have higher prevalence for PrEP use indicating networking and mixing pattern may be associated with the frequency and risk level of sexual behaviours, as well as other preventive measures such as PrEP and HIV testing [17]. Similar to another study, MSM of body image types related to habitual physical activity were more sexual health conscious [17]. Engaging in HIV risk behaviours, such as condomless sex, chemsex, and group sex, and perceiving to have an HIV-positive sex partner were associated with PrEP use. Our results showed that MSM at higher risk of HIV acquisition were aware of their risk and took additional preventive measures [20]. The higher perceived risk of HIV and STI among PrEP users was a supportive evidence. At the same time, it could also be the consequence of the phenomenon of risk compensation that PrEP users became more willing to engage in higher risk practices as PrEP reduced their HIV risk [21]. STI diagnosis could precipitate PrEP use for new users [22]; it could also be the result of engaging in higher risk sexual activities following PrEP use [23]. However, previous studies showed inconsistent results on whether PrEP would increase risk behaviours [24]. On the other hand, the dynamic pattern of sexual activities may result in the change in PrEP use. Our results indicated that some MSM who discontinued PrEP were less likely to engage in higher risk sexual activities. This echoed the results from a real-world implementation study that some MSM switched from daily to on-demand PrEP, and may as well stopped it altogether [5].

Vaccine was the most favoured option, as it represents a long-term effective preventive treatment, while the option of long-acting injectables was the second most favoured among other alternatives in the pipeline. HIV risk behaviours, particularly chemsex and group sex, and current PrEP use were associated with preferring injectables to other potential options. These factors were consistent with the findings in the literature [10]. During long chemsex sessions, adherence to oral tablets may be negatively affected by the drugs in some MSM [25]. Related lived experience shared by a PrEP user was being high at the time when PrEP should be taken, but he was only aware of the missed dose after sobering up [26]. On the other hand, the PROUD study results showed that daily PrEP adherence was not associated with chemsex [27]. Separately, efficacy was the only factor concerned by MSM who accepted long-acting injectables. This was somehow consistent with a previous qualitative study that safety, efficacy, and duration of protection were cited to be important factors to consider when switching from oral to injectable PrEP [28]. In our study sample, safety and dosage schedule was concerned by about 30% and they were not associated with acceptance rate of injectable PrEP. Intended long-term use of PrEP was also associated with injectable PrEP acceptance. For long-term users, injectable PrEP may offer a more convenient option to replace daily pill [29].

Previous studies showed that some MSM preferred a longer duration of at least six months to reduce the number of injections, or three months to align with regular blood tests [12]. One might therefore anticipate the acceptance rate of MSM would increase with the protective duration of the long-acting injectable drug. In our results, a bimodal distribution of the accepted protective duration was observed, highlighting that MSM accepted a monthly injection might not automatically accept a bimonthly one. The overwhelming preference of a monthly injection over a bimonthly one in our study was unexpected. One possible explanation could be a monthly schedule may seem to be more regular than a bimonthly one. The underlying reasons would require further studies. Even if we presume those accepted a shorter interval for injectable PrEP would accept a longer one, only 34% accepted a bimonthly regimen and 67% a biannually regimen.

With regard to the preferred PrEP service provider, those accepted injectable PrEP were more likely to favour public clinical services, including sexual health clinics, outpatient clinics, HIV services, and hospitals. Considering the type of service provider important was associated with all types of services. MSM preferring public hospitals were more concerned about privacy and potential labelling issues. This could be due to the volume of patients in the hospital diluting the potential stigma associated with PrEP [16]. Public hospitals may also have more resources to protect patients’ data privacy than community-based organisations. MSM preferring public outpatient clinics and hospitals were more concerned about the location and operating hours of the service. There were only a few sexual health clinics and HIV specialist clinics in Hong Kong (less than 10 combined), compared to over 70 general outpatient clinics distributed across the territory, which would be more accessible.

This study carries several limitations. The major limitation of a cross-sectional design is a causal relationship could not be established, as exposure and outcome were simultaneously assessed. Future longitudinal studies could be conducted to determine causative factors of PrEP and injectable PrEP use. Volunteer bias exists as the study participants were recruited using convenience sampling. As there would not be an exhaust list of MSM in the territory, probability sampling was not possible. Extrapolating the results to the community is therefore cautioned. As this study adopted a self-administered approach for data collection, self-report bias and recall bias may exist. We minimised recall bias by limiting the recall period to 6 months. In practice, one’s acceptability of different PrEP modalities could depend on multiple simultaneous factors. In this study these factors were assessed separately, further studies could be conducted to understand their preferences on the package of PrEP provision [30]. Relatively sensitive items such as STI diagnosis and chemsex history were only one of the covariates to be investigated instead of the primary outcome variable. The overall robustness remains largely unaffected. The self-administration and anonymous nature of the survey could reduce self-report and social desirability bias.

## Conclusions

Our study results provided an up-to-date landscape of PrEP use in the Hong Kong MSM community, as well as the acceptability of long-acting injectables as HIV PrEP should it become available. MSM at higher risk of HIV acquisition were aware of their risk level and therefore took additional preventive measures against HIV. Meanwhile, it cannot be ruled out that taking PrEP may have reinforced their risk behaviours. A proportion of PrEP users were willing to switch to a long-acting injectable. To facilitate the switch, they would need scientific evidence on its efficacy, given there is a safe and affordable product. The relatively low acceptance rate of a bimonthly or more frequent injection schedule may limit the switch or initiation of long-acting injectable PrEP. This prompted the need of future developments of a long-acting product providing a longer protection period for it to be widely acceptable in the MSM community.

## Supplementary Information

Supplementary Material 1

## Data Availability

The data that support the findings of this study are not openly available due to reasons of sensitivity and are available from the corresponding author upon reasonable request and with permission from The Chinese University of Hong Kong Survey and Behavioural Research Ethics Committee.

## Abbreviations

PrEP: Pre-exposure prophylaxis
MSM: Men who have sex with men
STI: Sexually transmitted infection
IQR: Interquartile range
OR: Odds ratio
CI: Confidence interval
CLAI: Condomless anal intercourse
aOR: Adjusted odds ratio
