# Supplementary material for: Utilisation of HIV pre-exposure prophylaxis and preferences of alternative long-acting modalities among men who have sex with men in Hong kong: a cross-sectional study
Source: BMC Public Health. 2025 Dec 26;26:361. doi: 10.1186/s12889-025-26025-5 (PMC12849621; doi:10.1186/s12889-025-26025-5)
Supplement: Supplementary file 1 — Supplementary Material 1 [file 12889_2025_26025_MOESM1_ESM.docx]

# S. Screening

S1. Are you aged 18 or above?
🌕 Yes 🌕 No [->EXIT]

S2a. What is your gender at birth?

🌕 Male 🌕 Female [->EXIT]

S2b. What is your self-identified gender?

🌕 Male 🌕 Female

S3. Have you ever had sex with another man before?
🌕 Yes 🌕 No [->EXIT]

S4. Did you live in Hong Kong and/or Macau most of the time (>50%) in the past 1 year?
🌕 Yes 🌕 No [->EXIT]

# A. Sociodemographics

A1. What is your year of birth? _______________

A2. Which of the followings best describes your ethnic identity?

🌕 Local (Hong Kong) Chinese 🌕 Local (Macau) Chinese

🌕 Mainland Chinese 🌕 Ethnic Chinese from other places 🌕 Southeast Asian

🌕 Local non-Chinese 🌕 Overseas non-Chinese 🌕 Others: __________

A3. Are you a local resident, i.e. holder of Hong Kong ID card or Macau ID card?

🌕 Yes, Hong Kong ID card holder 🌕 Yes, Macau ID card holder

🌕 Yes, Hong Kong and Macau ID card holder 🌕 No

A4. What is your education level?

🌕 Primary level or lower 🌕 Secondary level 🌕 Diploma or associate degree

🌕 Bachelor degree or higher

A5. Are you working or studying?

🌕 Full-time employment 🌕 Self-employed 🌕 Part-time employment / Freelancer

🌕 Student 🌕 Home-makers 🌕 Unemployed 🌕 Retired 🌕 Others: _____

A6. What is your current monthly income?

🌕 < HK$5,000 🌕 HK$5,001 - 15,000 🌕 HK$15,000 - 30,000

🌕 HK$30,001 - 50,000 🌕 > HK$50,000

A8. Which of the (combination of) followings best describes your appearance? (select all that apply)

 slim / slim fit  fit / lean / lean-toned  muscular / macho

 meaty  bear  chubby / fat

 feminine / sissy  business suit  decent

 sporty  mature  manly

 tanned  cute  hairy  Others (please specify): ______

# C. Your sexual history in the past 6 months

C1a. Have you used any channel to seek **new male sex partners** in the past 6 months?

○ Yes ○ No

C2. How often were you a bottom for anal sex with male sex partners in the past 6 months?

○ Usually top ○ Half bottom half top ○ Usually bottom ○ No anal sex [=> C5]

C3. How often did you use a condom when having anal sex with a male partner in the past 6 months?
○ Never ○ less than a half of the time ○ more than a half of the time ○ every time [=> C5]

C4. How often was PrEP used when having condomless anal sex with a male partner in the past 6 months?
○ Never ○ less than a half of the time ○ more than a half of the time ○ every time

C5. Did you have sex with the following type(s) of male sex partners in the past 6 months?

 Non-regular sex partner (one night stand without money exchange)

 Regular sex partner (Multiple sex acts without money exchange and emotional attachment)

 Boyfriend (regular sex partner with emotional attachment)

 Sex partner who get paid for sex (You pay to have sex)

 Sex partner who paid for sex (You get paid for sex)

 I did not have sex with a male partner in the previous 6 months

C6. Have you/your male sex partner used entertaining drugs before/when having sex (chemfun/chemsex) in the past 6 months?

○ Yes, I have ○ Yes, my partner(s) have ○ Yes, my partner(s) and I have ○ No [=>C7]

○ I did not have sex with a male partner in the previous 6 months [=>C7]

C6b. What kind of recreational drug(s) have you/your male sex partner used? (select all that apply)

 Poppers/RUSH Viagra / Levitra / Cialis  Ketamine Methamphetamine / Ice

GHB 0 capsule Marijuana/ Cannabis Others (please specify): _____

C7. Did you do the following items in the past 6 months? (select all that apply)

 Engaging in sex involving more than two persons (e.g. 3P)  Having sex with female

 Sharing sex toys without a new condom or sharing enema equipment

 Fisting (recipient)  Sharing injecting drug equipment ○ None of the above

C8. How likely do you think you will have condomless anal sex in the coming 6 months?

○ Definitely not ○ Unlikely ○ Likely ○ Definitely

C9. What is your perceived risk of HIV infection?

○ High risk ○ Medium risk ○ Low risk ○ No risk ○ Not applicable (I am living with HIV)

C10. Do you think any of your male sex partners have been infected with HIV?

○ Definitely yes ○ Probably yes ○ Probably no ○ Definitely no

C11. In general, what is your perceived risk of sexually transmitted infections other than HIV?

○ High risk ○ Medium risk ○ Low risk ○ No risk

# D. HIV/STI testing history

D1. What is your frequency of **HIV testing**?

○ Once per 3 months or less ○ Once per half year ○ Once per year ○ Once per two year

○ Once per more than two years ○ Only tested once ○Never [=> D3a]

D2a. When did you last test for HIV? Year ___ Month ______

D2c. If tested, do you know the results of your last HIV test?

○ Yes, infected ○ Yes, not infected [=> D3a] ○ Don’t know/forgot [=> D3a]

D2d. Which year were you diagnosed with HIV? Year _______

D3a. What type of STI symptoms have you ever experienced? (select all that apply)

○ No symptom  Discharge  Dysuria  Ulcer  Growth, including wart

 Rash  Blister  Others : _________________

D3b. Have you experienced any STI symptoms by the following body site(s)? (select all that apply)

○ No STI symptoms  Genital or urinary  Anal  Mouth Skin  Others: ______

D4. What is your frequency of **STI testing**?

○ Once per 3 months or less ○ Once per half year ○ Once per year ○ Once per two year

○ Once per more than two years ○ Only tested once ○Never [=> D5a]

D4a. When did you last tested for STI?

Year ___ Month ______

D4b. What STI did you last test for? (select all that apply)

 Syphilis  Gonorrhoea  Chlamydia  Monkeypox  Mycoplasma
 Hepatitis C  Genital wart/HPV  Genital herpes  Others, specify: __________

D5a. Have you ever been diagnosed with any STI? (select all that apply)

 I have never been diagnosed with an STI

 Syphilis  Gonorrhoea  Chlamydia  Monkeypox  Mycoplasma
 Hepatitis C  Genital wart/HPV  Genital herpes  Others, specify: __________

D5b. Have you been diagnosed with any STI in the past 6 months?

○ Yes ○ No

# E. Your awareness of PrEP

E1a. How familiar are you with the idea of PrEP

🌕 I know PrEP extremely well 🌕 I have general understanding of PrEP

🌕 I only have vague idea about PrEP 🌕 I have no idea what PrEP actually is

E1b. How effective do you think PrEP is for preventing sexual transmission of HIV from an infected partner to a HIV-negative person?

🌕 almost perfect protection at about 100% 🌕 very effective at over 75%

🌕 moderately effective at around 50% 🌕 mildly effective at below 50% 🌕 Ineffective

E2. Have you ever used PrEP

🌕 Yes 🌕 No [=> F1]

E3. Are you currently on PrEP?

🌕 Yes I am continuing with daily PrEP 🌕 Yes I am continuing with on-demand PrEP

🌕 I am no longer on PrEP

**F. Your views about PrEP**

F1. There are two PrEP approaches; the first is taking a daily tablet, and the alternative approach is PrEP on demand, which involves taking 2 tablets 2-24 hours before sex and then 1 tablet each at 24 hour and 48 hour (the 2+1+1 regimen) subsequently. If access is not a problem, what is your preferred mode of PrEP?

🌕 Daily tablet 🌕 On demand (2+1+1)

🌕 Daily alternating with on demand, to tie in with sexual activity pattern

🌕 Would not take PrEP [=> F6]

F2. The reason(s) why you prefer this mode is/are: (select all that apply)

 Easier to remember the time to take pills

 Simpler regimen  Flexible regimen  Less pills are required to be taken

 More adapted to sex pattern  More confident about ensuring protection

 Consistent with sex partner's mode of dosage  Less disruption to daily life

 Less side-effects  Others : ________________

F3. If PrEP requires a fee for service, how much are you prepared to pay for PrEP per month on a regular basis, on the antiretroviral medicine alone?

🌕 Won’t use PrEP if it requires payment

🌕 < HK$500 🌕 HK$501 – 1,000 🌕 HK$1,001 – 2,000

🌕 HK$2,001 – 5,000 🌕 HK$5,000 or above

F4. What would you anticipate to be the source of money to pay for PrEP if it becomes available as a fee-for-service? (select all that apply)

 Self-payment  Sex partner(s)  Boyfriend(s)/Girlfriend(s)/Spouse

 Family member(s)  My medical insurance if covered

 My company's health policy if covered  Others : ____________  Will not take PrEP

F5. How long will you expect yourself to be on PrEP if you start taking it today?

🌕 Only 1- 3 months then stop

🌕 1- 3 months then decide later

🌕 3-12 months then decide later

🌕 1-2 years then decide later

🌕 Prolonged use for over 2 years

F6. What are the factors that would affect your decision of considering taking PrEP? (select all that apply)

 Efficacy  Price  Dose regimen  Side-effect

 Service organisation  Service location  Service time  Privacy

 Embarrassment or stigma related to  Others : _______________

**G. New generation of PrEP**

G1. Different new modes of PrEP delivery are now being tested. Which of the followings are you looking forward to? (select all that apply)

 injectable drug  vaccine  Implant or transdermal device

 rectal gel and enema  none of the above  Would not take PrEP

G2. If there is a longer acting antiretroviral which may increase the interval between two doses for PrEP so that oral daily use is not necessary. What is your preferred interval of 2 doses of oral PrEP ?

🌕 weekly 🌕 2-weekly 🌕 monthly 🌕 2-monthly 🌕 3-monthly 🌕 remain daily

🌕 other: ____________ 🌕 Would not take PrEP

G3. If there is an injectable drug for PrEP, what is the shortest interval between two doses do you accept?

🌕 weekly 🌕 2-weekly 🌕 monthly 🌕 2-monthly 🌕 3-monthly 🌕 6-monthly

🌕 yearly 🌕 Will not consider injectable drug 🌕 Would not take PrEP

G4. If PrEP doses can be taken at longer interval, what is your preferred mode of antiretroviral administration?

○ oral ○ injection ○ implant or transdermal device ○ longer interval antiretroviral not preferred

○ Would not take PrEP
